# *Astragalus membranaceus* extract reduces functional knee joint pain: a randomized, double-blinded, placebo-controlled trial

**DOI:** 10.3389/fpain.2025.1595957

**Published:** 2025-10-29

**Authors:** Lorenzo Lippi, Alessio Turco, Girish H. Rudrappa, Stefano Moalli, Alessandro de Sire, Marco Invernizzi

**Affiliations:** ^1^ Department of Health Sciences, University of Eastern Piedmont “A. Avogadro”, Novara, Italy; ^2^ Department of Scientific Research Campus LUdeS Lugano (CH), Off-Campus Semmelweis University of Budapest, Budapest, Hungary; ^3^ Rajalakshmi Hospital & Research Center, Bangalore, India; ^4^ Physical Medicine and Rehabilitation Unit, Department of Medical and Surgical Sciences, University of Catanzaro “Magna Graecia”, Catanzaro, Italy; ^5^ Research Center on Musculoskeletal Health, MusculoSkeletalHealth@UMG, University of Catanzaro “Magna Graecia”, Catanzaro, Italy; ^6^ Translational Medicine, Dipartimento Attività Integrate Ricerca e Innovazione (DAIRI), Azienda Ospedaliera SS. Antonio e Biagio e Cesare Arrigo, Alessandria, Italy

**Keywords:** joint pain, knee, *Astragalus membranaceus*, dietary supplements, phytotherapy, rehabilitation

## Abstract

**Introduction:**

Concerns regarding the side effects of pharmacotherapy in the management of joint pain have led to increased interest in dietary supplements. *Astragalus membranaceus* root extract (AME) has been proposed as an alternative approach to relieving knee joint pain. The present study evaluated the efficacy and safety of a standardized AME in patients with functional knee joint pain.

**Methods:**

A double-blind, randomized controlled trial was conducted with 90 adults (18–60 years of age) from Rajalakshmi Hospital and Research Center, Karnataka, India. Participants were randomly assigned to receive either 480 mg of AME (*n* = 45) or placebo (*n* = 45) for 28 days. The primary outcome was knee pain reduction, which was assessed using a visual analog scale (VAS) after a 6-min walk test. Secondary outcomes included the Western Ontario and McMaster Universities Osteoarthritis Index (WOMAC), Stair Climb Test (SCT), knee range of motion (ROM), and treatment compliance, evaluated at baseline and at follow-up on days 5, 14, and 28. Patient satisfaction and safety were also assessed.

**Results:**

The AME group exhibited a significant 30% reduction in knee pain (*p* < 0.0001), with mean VAS scores dropping from 6.7 ± 0.5 to 1.2 ± 0.6. Significant improvements were observed in the WOMAC, SCT score, and ROM (*p* < 0.0001). Patient satisfaction was higher in the active-treatment group, and no serious adverse events were reported.

**Discussion:**

AME was a safe and effective alternative for the management of knee joint pain and merits further longer-term investigation.

**Clinical Trial Registration:**

https://ctri.nic.in/Clinicaltrials/pmaindet2.php?EncHid=OTE3MTU=&Enc=&userName=, identifier CTRI/2023/09/057317.

## 1 Introduction

Joint issues represent highly prevalent conditions affecting 35 million individuals worldwide, with significant implications for activities of daily living (ADL) and health-related quality of life (1–3). While it often manifests in individuals with underlying conditions, knee pain may also be related to biomechanical imbalances, muscular weakness, overuse activities, or lifestyle factors such as sedentary behavior (4–7). Furthermore, knee pain may significantly affect physical functioning and mobility, with negative implications for overall well-being (8).

Traditional approaches to the management of knee pain have mainly focused on symptoms, and include pharmacological and non-pharmacological interventions (9). Nonsteroidal anti-inflammatory drugs (NSAIDs) are first-line therapies for pain relief. However, the prolonged use of NSAIDs or analgesics is associated with a high risk for potential adverse effects, including gastrointestinal complaints and cartilage damage (10). Consequently, the potential side effects associated with pharmacotherapy underscore the need for alternative strategies that offer effective symptom relief without affecting long-term health (11).

Dietary supplements have emerged as promising adjunctive therapies for managing knee issues in healthy individuals, offering a natural—and, potentially, safer—alternative to conventional medications (12). Several nutraceuticals have been proposed to improve joint health, including collagen, glucosamine sulfate, chondroitin, omega-3 fatty acids, vitamin D, probiotics, curcumin, Boswellia, and other botanical extracts (13–16). These supplements often contain ingredients with anti-inflammatory, antioxidant, and cartilage-supporting properties, which offer natural alternatives to conventional medications (16).

In this context, astragalus extract derived from the roots of *Astragalus membranaceus* (Fisc.) Bunge (Fabaceae) is widely used in Traditional Chinese Medicine (TCM) because of its multifaceted health benefits and high safety profile (17). Recently included in the European Pharmacopoeia, *A. membranaceus* root extract(s) (AME) has demonstrated various biological functions, including immunomodulatory and hepatoprotective effects (18–20). Interestingly, the primary active components of AME include flavonoids, saponins, and polysaccharides, which exhibit anti-inflammatory and anti-arthritic properties (17, 21).

The bioactive components of *A. membranaceus*, such as flavonoids, polysaccharides, and saponins [particularly astragaloside (AST) IV], have multifaceted effects on the oxidative and inflammatory pathways linked to joint degeneration (22). By blocking the activation of nuclear factor-kappa B (NF-κB), AST IV has been demonstrated to downregulate the expression of important pro-inflammatory cytokines, including tumor necrosis factor-alpha (TNF-α), interleukin (IL)-1 beta (IL-1β), and IL-6 (23). Inhibition of NF-κB signaling may reduce cartilage matrix degradation, chondrocyte apoptosis, and synovial inflammation (23).

In parallel, Astragalus polysaccharides have demonstrated immunomodulatory activity by regulating T-cell responses and macrophage polarization, thereby creating an overall anti-inflammatory environment (24). Furthermore, these polysaccharides have been linked to the inhibition of matrix metalloproteinases (MMP-1, MMP-3, and MMP-13), which mediate the breakdown of collagen and proteoglycans in articular cartilage (25) and helps to maintain the integrity of the extracellular matrix.

The enhancement of the endogenous defense system is one way in which *A. membranaceus* exerts its antioxidant potential. Its phytochemical components have been shown to specifically upregulate antioxidant enzymes, such as glutathione peroxidase, catalase, and superoxide dismutase (26). An important pathological characteristic of both inflammatory and degenerative joint conditions is the oxidative stress-induced damage to chondrocytes and synovial tissues, which can be mitigated by neutralizing reactive oxygen species (ROS).

These mechanisms suggest that AME synergistically modulates immune, oxidative, and catabolic pathways to produce chondroprotective and anti-inflammatory effects, offering a tenable biological justification for its use in the treatment of joint disorders (27).

Interestingly, Maresca et al. (17) assessed the effects of hydroalcoholic extract administration on joint pain in rats and reported significant benefits in terms of pain severity. Although this preclinical evidence supports the efficacy of AME supplementation in the management of joint health, to the best of our knowledge, its effects on human joint health have not been widely explored to date. Moreover, although AME supplementation has been studied in other clinical conditions, including cancers and cardiac and metabolic disorders (28–30), its impact on patients with knee pain remains unclear and, moreover, no randomized controlled trial addressing this topic is currently available.

As such, the present randomized, double-blinded, placebo-controlled trial aimed to assess the effects of the standardized hydroalcoholic extract obtained from *A. membranaceus* root (Axtragyl, Giellepi S.p.A., Milan, Italy) on pain severity, joint function, and mobility, as well as safety among individuals affected by functional knee pain.

## 2 Materials and methods

### 2.1 Participants

This double-blind, randomized, placebo-controlled trial adhered to the CONSORT guidelines (31), and the study protocol was registered with the Clinical Trial Registry of India (CTRI/2023/09/057317).

A consecutive series of patients referred to the Rajalakshmi Hospital and Research Center of Karnataka, India, were assessed. The inclusion criteria were as follows: 18–60 years of age; functional knee joint pain (not linked to acute injury or chronic disease); pain intensity during activities, such as ADL and/or low-intensity physical activity, between moderate and severe [visual analog scale (VAS) 4–7]; body mass index (BMI) <27 kg/m^2^; no abnormalities on electrocardiographic screening; no abnormalities on creatine kinase screening; and informed written consent to adhere to the study protocol.

The exclusion criteria were as follows: use of other herbal supplements, anti-inflammatory, or analgesic drugs during the study period, potentially interfering with the investigational product and study outcomes; occurrence of soft tissue injuries in the upper or lower limbs; severe medical or psychological conditions, chronic inflammation, or infections; joint surgery within the previous 3 months; pregnancy, lactation, or lack of effective contraceptive methods among females of childbearing potential; and participation in another clinical study within 30 days before the start of this study.

The trial protocol was approved by the Institutional Review Board and adhered to the principles of the Declaration of Helsinki (32) and relevant national and international regulatory requirements. Written and oral information regarding the study procedures was provided to all participants. Before the screening evaluation, each participant was informed about the purpose of the clinical trial, including the possible risks and benefits, and informed consent was documented in the participants’ charts. Participants were informed about the potential adverse events associated with the treatment, with each providing informed written consent before entering the study or initiating any study-related procedures.

### 2.2 Intervention and procedures

At baseline, 124 participants were screened for demographic characteristics and underwent physical examination, including vital signs, and medical and medication histories. Laboratory investigations were performed, including complete blood count (CBC), serum glutamic-pyruvic transaminase (SGPT), serum glutamic-oxaloacetic transaminase (SGOT), creatinine kinase, urine for pregnancy, and serum creatinine level. Knee pain using the VAS was assessed after a 6-min walk test. Any concomitant medications were recorded.

After all baseline assessments, participants who fulfilled the eligibility criteria were enrolled and randomly assigned by computer-generated simple randomization into two groups with a 1:1 allocation. The randomization sequence used a block randomization method with a fixed block length of 4 participants, ensuring an equal distribution of participants between the groups within each block. The randomization list was computer-generated using SAS version 9.4 (SAS Institute, Cary, NC, USA) by an independent operator who was not involved in any other part of the study. Blinding was ensured because both participants and study personnel were unaware of the group allocation. Additionally, a placebo (i.e., control) that resembled every study intervention was used. Sealed envelopes were used, with each indicating the randomization number and specific test product.

Participants in the active group were orally administered 2 capsules containing 240 mg AME per day, whereas subjects in the placebo group received two capsules containing 240 mg placebo daily for 28 consecutive days. Both the active and placebo capsules were tasteless, odorless, and identical in shape, color, and dimensions.

The investigational product (Axtragyl, Giellepi S.p.A., Milan, Italy) is a proprietary hydroalcoholic extract obtained from A. membranaceus root, standardized to the main phytochemical components (including terpenes, flavonoids, and typical astragalus polysaccharides) found in the plant (33).

### 2.3 Outcome measures

Primary and secondary endpoints were evaluated at baseline (T0), and after 5 ± 2 (T1), 14 ± 2 (T2), and 28 ± 2 (T3) days. The primary endpoint was improvement in knee pain according to the VAS after a 6-min walk test between the active and placebo groups from baseline to the end of treatment. The secondary endpoints included improvement in knee function and discomfort, assessed according to the following clinical outcome measures:

- Western Ontario and McMaster Universities Osteoarthritis Index (WOMAC) (34): This scale is a questionnaire consisting of 24 items designed to assess the symptoms and physical function of individuals with hip or knee pain. The index comprises 3 main domains: pain (5 items), stiffness (2 items), and physical function (17 items). Participants rated their experiences on a scale ranging from 0 (no symptoms or difficulty) to 10 (extreme symptoms or difficulty).
- Stair Climb Test (SCT) (35): The SCT a performance test used to evaluate lower limb strength, endurance, and mobility. Participants ascended and descended a set of 10 stairs as quickly and safely as possible, with time measured by the operator.
- Knee range of motion (ROM): A goniometer was used to measure flexion and extension angles of the knee joint with the participant positioned supine. Knee flexion was measured by asking the participant to bend the knee and slide the heel as far as possible toward the buttocks. Subsequently, to measure knee extension, participants were asked to slide their heels, actively extend the knee, and bring it to maximum extension. Normal knee ROM is typically 0°–135° of flexion. These assessments aid in diagnosing joint issues, tracking the progress of rehabilitation, and evaluating interventions.

Other secondary endpoints included safety and tolerability assessments of the different markers at baseline and end of the study period. Physical examination, vital signs, and laboratory investigations, including CBC, SGPT, SGOT, creatinine kinase, and serum creatinine, were considered for safety evaluations. C-reactive protein (CRP) level was assessed as a marker of inflammation.

Tolerability and satisfaction were assessed using a self-assessment questionnaire and five-point Likert scale (36).

### 2.4 Statistical analysis

Sample size was calculated using G*Power3 software based on the primary endpoint. With a statistical power (1–β) of 90% and a significance level (α) of 0.05 (5%), the calculated sample size was 43 for each group. However, a drop-out rate of 5% was projected, resulting in an overall sample size requirement of 90 for the study, with 45 in each group. The level of significance (two-sided) was set at 5% with a power of 90%.

Statistical analyses were performed using Prism version 9.0 (GraphPad Inc., San Diego, CA, USA). Continuous variables are expressed as mean ± standard deviation, while categorical variables are expressed as number and percentage. Non-parametric tests were chosen due to the non-normal distribution of variables, which was assessed using the Shapiro–Wilk test. According to the intention-to-treat principle, the participants were analyzed based on their originally assigned groups to account for missing data and preserve randomization integrity. Intragroup differences were evaluated using the Wilcoxon signed-rank test, and differences between groups were assessed using the Mann–Whitney U test. Descriptive statistics were used to summarize the adverse effects of the treatment.

## 3 Results

**Figure 1:**
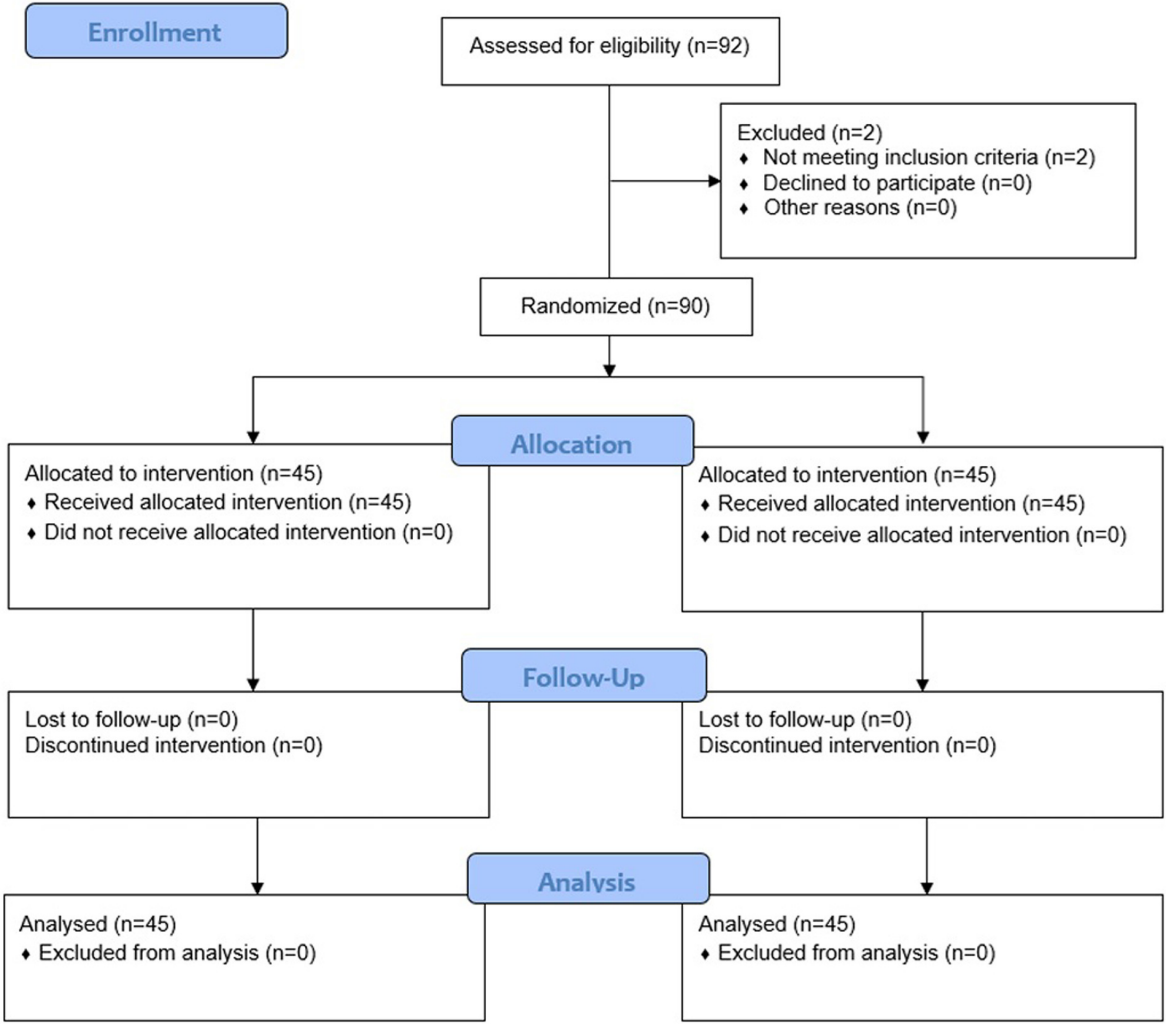
Consort study nflow chart.

Of 92 individuals assessed for eligibility, 2 were excluded because they did not fulfil the inclusion criteria; as such, 90 were randomly assigned to either the intervention group [AME (Axtragyl (*n* = 45)] or control group [placebo (*n* = 45)]. The CONSORT 2010 flow-diagram illustrating the enrolment and allocation processes is presented in Figure 1. The active group included 23 females and 22 males, with a mean age of 48.2 ± 6.30 years and a mean BMI of 23.67 ± 2.16 kg/m^2^. The placebo group included 22 females and 23 males, with a mean age of 47.4 ± 4.98 years and a mean BMI of 23.68 ± 1.93 kg/m^2^. A comprehensive summary of the baseline characteristics of both groups is presented in Table 1. No dropouts were reported during the study period and all participants completed the outcome analysis without protocol deviations.

**Table 1:** Anamnestic, demographical, and clinical characteristics of the study population. Continuous variables are expressed as means ± standard deviations, categorical variables are expressed as counts (percentages).

Sample characteristics	Axtragyl® (*n* = 45)	Placebo (*n* = 45)
Age (years)	48.2 ± 6.30	47.4 ± 4.98
Gender (female/male)	23/22	22/23
Weight (kg)	66.1 ± 6.45	63.1 ± 8.77
Height (cm)	167.2 ± 5.40	162.8 ± 7.01
BMI (kg/m^2^)	23.67 ± 2.16	23.68 ± 1.93
VAS	6.7 ± 0.5	6.7 ± 0.5

BMI, body mass index; VAS, visual analogue scale.

**Figure 2:**
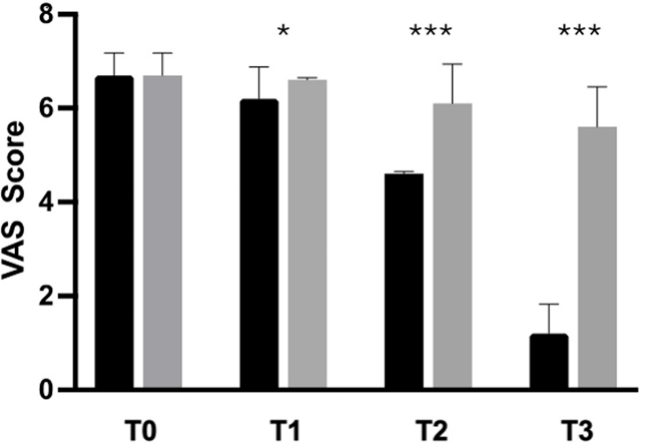
Improvement of knee pain (VAS) from baseline to the end of the study. Black: Axtragyl®; grey: placebo. T0: Baseline; T1: day 5; T2: day 14; T3: day 28. *p < 0.05 vs. placebo; ***p < 0.0001 vs. placebo.

Analysis of the primary outcome measure revealed that 88.9% of participants in the AME group experienced a 30% reduction in knee pain after the activity, with a statistically significant difference between the groups (*p* < 0.0001). Moreover, the mean VAS score in the active-treatment group decreased, from 6.7 ± 0.5 at baseline, to 1.2 ± 0.6 after 28 days, with significant differences between groups at each time point (Figure 2). In the placebo group, VAS score decreased, from 6.7 ± 0.5 at baseline, to 5.6 ± 0.9 after 28 days.

**Figure 3:**
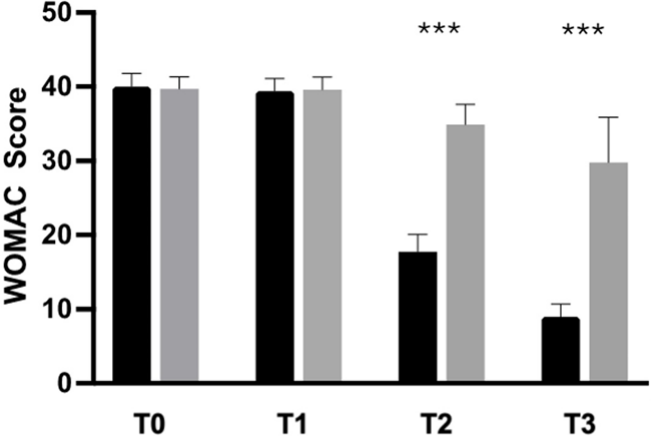
Improvement of WOMAC from baseline to the end of the study. Black: Axtragyl®; grey: placebo. T0: Baseline; T1: day 5; T2: day 14; T3: day 28. ***p < 0.0001 vs. placebo.

**Figure 4:**
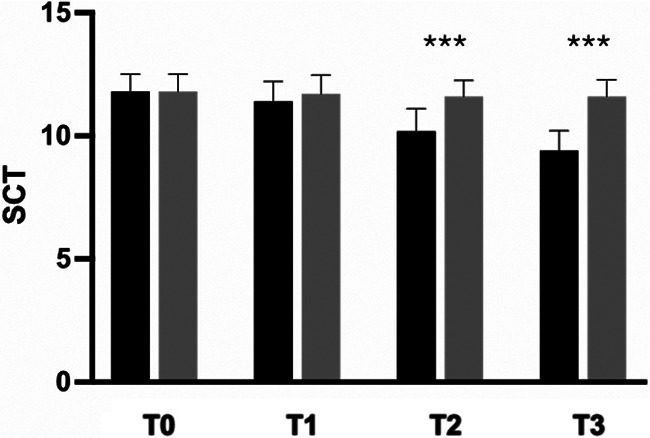
Improvement of SCT from baseline to the end of the study. Black: Axtragyl®; grey: placebo. T0: Baseline; T1: day 5; T2: day 14; T3: day 28. ***p < 0.0001 vs. placebo.

The secondary outcome analysis revealed significant changes in WOMAC score in the active-treatment group, indicating an improvement in knee function from baseline (Figure 3). Statistically significant differences were observed between the groups on days 14 and 28 (*p* < 0.0001). Similarly, SCT results revealed significant improvement over placebo in the active-treatment group on days 14 and 28 (*p* < 0.0001) (Figure 4).

**Figure 5:**
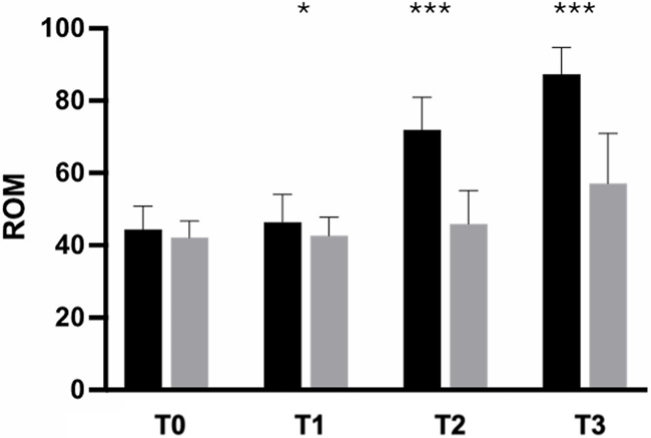
Improvement of ROM from baseline to the end of the study. Black: Axtragyl®; grey: placebo. T0: Baseline; T1: day 5; T2: day 14; T3: day 28. *p < 0.05 vs. placebo; ***p < 0.0001 vs. placebo.

In contrast, no significant differences in either the WOMAC score or SCT results were observed in the between-group analysis after 5 days of supplementation. Participants in the active-treatment group exhibited superior improvement in pain-free ROM compared with those in the placebo group, with statistically significant differences at each time point (*p* < 0.0001) (Figure 5).

In addition, the patient satisfaction assessment revealed a higher satisfaction rate in the active therapy group than in the placebo group (*p* < 0.0001). Overall tolerability was rated as “Excellent” by 91.1% of participants in the active-treatment group and 82.2% in the placebo group. The remaining participants (8.9% in the active-treatment group and 17.8% in the placebo group) rated it as “Good”, with no reports of “Fair” or “Poor” in either group.

The mean adherence rates in the active-treatment and placebo groups was 100%, with no significant intergroup differences.

In this study, 5 non-serious adverse events were observed, 4 of which were in the active-treatment group and 1 in the placebo group. These were minor events (gastrointestinal complaints and nausea) that resolved within 1 day without any medication. Each event was classified as unrelated to the study treatment, based on a clinical assessment performed by blinded medical personnel. All adverse events resolved without any complications.

There were no abnormal changes in physical examination or vital signs throughout the study. Hematochemical parameters were within normal limits from baseline to the end of the study.

## 4 Discussion

In recent years, increasing attention has been devoted to the benefits of dietary supplements in healthy individuals, with the aim of promoting joint health and improving mobility, physical functioning, and overall well-being. Although several studies have highlighted the positive effects of different nutraceuticals on the skeletal muscle system, the effects of *A. membranaceus* on human joint health remain largely unexplored.

As such, this randomized controlled trial provides promising data supporting the potential role of the hydroalcoholic extract of *A. membranaceus* root in managing functional knee pain in healthy participants. Interestingly, the primary outcome assessment demonstrated a significant reduction in pain severity, according to the VAS, in the active-treatment vs. the placebo group, with 88.9% of participants experiencing a clinically meaningful (i.e., 30%) improvement. In contrast, 7 participants in the placebo group recorded only slight and no clinically relevant pain relief.

The results of our investigation are consistent with those of previous preclinical studies (17, 33), reporting that a single administration of AME significantly reduced joint pain, joint inflammatory infiltrate, and plasma levels of pro-inflammatory cytokines in an experimental rat model of arthritis. Although these results were obtained from studies involving animal models, they provide insights for further clinical research investigating the treatment of human joint discomfort.

Moreover, secondary outcome measures further supported the beneficial effects of supplementation with the investigational product in terms of knee pain, stiffness, and physical function, as assessed using the WOMAC. In particular, the WOMAC assessment revealed a significant improvement in the AME group vs. the placebo group, indicating enhanced overall joint health and function. Similarly, performance measures, such as SCT and knee ROM, demonstrated superior outcomes in the active-treatment group, underscoring the positive impact of treatment on lower limb strength, mobility, and flexibility. These data are consistent with previous preclinical studies suggesting the positive effects of AME on joint pain and function (17). In particular, it has been reported that its components, including isoflavones, flavonoids, and saponins, may have intriguing implications in the management of joint damage and inflammation (17, 20, 21). Huh et al. (21) identified a controversial effect of *A. membranaceus* on human subchondral osteoblasts from both healthy individuals and those diagnosed with osteoarthritis. Although the isoflavones found in AME may increase osteolytic activity under normal conditions, they interfere with osteoblast metabolism, restricting subchondral remodeling related to cartilage disorders (21). In contrast, flavonoids in AME are mainly composed of calycosin, which reduces synovial fluid production and mitigates subchondral bone damage (37).

Additionally, astragalus saponins [such as astragaloside(s) (AST)] have potential implications in immunomodulation at the joint level, with evidence suggesting a reduction in plasma levels of TNF-α and IL-1β, and alleviation of inflammation in knee synovial tissue in rat models (38, 39). Moreover, articular diseases are frequently characterized by oxidative stress, which results in increased production of local ROS production and inflammatory responses (13, 40, 41). In contrast, isoflavones, flavonoids, and saponins exhibit significant activity against oxidative imbalances (42), with positive implications for their antioxidant and analgesic effects. Moreover, experimental findings reported in the literature clearly demonstrate that AME, through its specific active components (such as AST and flavonoids), exert their anti-inflammatory—and, potentially pain-relieving—effects by modulating key inflammatory pathways and reducing various inflammatory biomarkers.

An AME was found to decrease the serum levels of TNF-α and IL1-β in a dose-dependent manner in a rat model of adjuvant-induced arthritis (AIA) (43).

Similar results were found in a different study performed in a mouse model of osteoarthritis, as well as in patient-derived chondrocytes (44). In particular, AST induced a dose-dependent inhibition of inflammatory mediators, including IL-6, TNF-α, nitric oxide (NO), and prostaglandin E2 (PGE2). AST inhibits the activation of NF-κB signaling, a central pathway in inflammatory responses.

A previous study (37) focused on the effects of AST IV, one of the most active secondary metabolites of *A. membranaceus* root, on the inflammatory response in a rat AIA model. The authors found that treatment with AST suppressed joint inflammation by inhibiting IL-1β, TNFα, and NO production in AIA model rats. Macrophages are the main targets of AST. Moreover, the treatment exerted a protective effect against IL-1β-induced damage of cartilage, and sustained both proteoglycan synthesis and chondrocyte proliferation. Collectively, these findings suggest that AST may be involved in the prevention of IL-1β-induced joint inflammation and cartilage destruction.

Several key differences emerged when comparing AME with other dietary supplements commonly used for joint health. Collagen supplementation is frequently used in joint pain management due to its potential role in supporting joint structure and function by providing essential building blocks for cartilage repair and maintenance (45). While collagen has yielded promising results for improving joint pain and function in several studies, evidence remains uncertain due to variability in outcome measures, differences in the tested formulations, and heterogeneous study populations (46, 47). Similarly, chondroitin sulfate is another widely used supplement that may exert its effects by enhancing cartilage hydration and resilience, thereby mitigating joint pain and stiffness (48). Similar to collagen, chondroitin sulfate has demonstrated efficacy, particularly when used in combination with glucosamine (49, 50). However, conflicting results and methodological limitations in clinical trials have raised questions regarding overall effectiveness in individuals without joint degeneration (51, 52).

Unlike collagen and chondroitin sulfate, which primarily focus on providing structural support to joints, AME may target both anti-inflammatory and antioxidant pathways (17, 53). In addition, the supplement tested in the present trial demonstrated statistically significant fast action within merely 5 days, as evidenced by improvements in VAS scores and ROM, in contrast to other natural compounds requiring longer periods to achieve clinically perceptible relief (46, 47, 51, 52). In this context, the diverse array of bioactive compounds found in the astragalus root phytocomplex of the investigational product (i.e., Axtragyl) may synergistically contribute to its health benefits, offering a holistic approach to joint health (54).

Furthermore, the high adherence to treatment recorded in our trial reflects the tolerability and acceptability of the treatment among the study participants, whereas the low dropout rate may be related to rapid relief. Finally, the low incidence of adverse events underscores the safety profile of the investigational supplement as a well-tolerated intervention for knee disorders.

In recent years, increasing research has focused on the benefits of combined rehabilitation, including physical therapy and nutritional intervention with dietary supplements, to enhance rehabilitation outcomes (55, 56). By modulating inflammatory pathways and oxidative stress, the investigational supplement may synergize with rehabilitation or therapeutic exercises, creating a more favorable physiological environment for tissue repair and remodeling and optimizing outcomes in individuals experiencing knee pain (57).

This was the first double-blind, placebo-controlled trial to assess the effects of AME on knee pain, providing clinical evidence supporting its safety and effectiveness in reducing pain intensity and increasing physical joint functioning. These findings may have positive implications for quality of life because knee joint issues have been associated with impaired independence in ADLs and reduced overall well-being (8, 58, 59). Despite these considerations, this study had several limitations. The lack of follow-up does not provide insight into the long-term effects or durability of the pain relief provided by the intervention. Thus, future studies with extended follow-up periods are warranted to evaluate the sustainability of these therapeutic benefits. Additionally, although the sample size was adequate to detect significant differences in the primary and secondary outcomes, further multicenter studies with larger cohorts may provide additional insights into the generalizability of these findings. An additional limitation was the absence of inflammatory biomarker measurements.

However, further research is required to explore these inflammatory markers in greater detail, investigate the relevant inflammatory pathways more comprehensively, and elucidate the underlying mechanism(s) associated with the observed clinical effects. Notably, the current study included healthy adults experiencing functional knee pain, which may differ substantially in pathophysiology and symptomatology from the degenerative joint conditions typically encountered in the elderly population. In patients with osteoarthritis, factors such as cartilage degradation, osteophyte formation, and chronic synovial inflammation may modulate symptom severity and treatment responsiveness. Therefore, although the anti-inflammatory and antioxidant properties of AME may theoretically confer benefits against osteoarthritis, these effects need to be empirically tested in populations with clinically and radiologically confirmed joint degeneration. Future clinical trials are needed to evaluate whether the findings of this study can be extrapolated to older individuals or to those with varying degrees of structural joint damage to establish the efficacy across a broader patient spectrum.

## 5 Conclusions

To the best of our knowledge, this is the first double-blind randomized controlled trial to provide insights into the role of AME in preserving joint health. Results of this study support its efficacy and safety as a promising dietary supplement for managing functional knee pain in healthy individuals. Further research exploring its long-term effects, mechanism(s) of action, and potential synergies with conventional treatments may be useful for improving the current understanding of its role in long-term joint pain management.

## Data Availability

Baseline characteristics of the study population are provided as Supplementary Material. The full dataset is the property of Giellepi S.p.A., which retains all rights regarding data access and distribution. The raw data supporting the conclusions of this article will be made available by the Giellepi S.p.A upon request. The company is solely responsible for data stewardship, including its availability, integrity, and governance.
